# Designing Smart Objects to Support Affording Situations: Exploiting Affordance Through an Understanding of Forms of Engagement

**DOI:** 10.3389/fpsyg.2018.00292

**Published:** 2018-03-12

**Authors:** Chris Baber

**Affiliations:** School of Engineering, University of Birmingham, Birmingham, United Kingdom

**Keywords:** affordance, smart objects, animate objects, interactivity, forms of engagement

## Abstract

In this paper I consider how the concept of “affordance” has been adapted from the original writings of Gibson and applied to interaction design. I argue that a clear understanding of affordance shifts the goal of interaction design from one of solely focusing on either the physical object or the capabilities of the person, toward an understanding of interactivity. To do this, I develop the concept of Forms of Engagement, originally proposed to account for tool use. Finally, I extend this concept to interacting with modified tangible user interfaces, or “animate objects.” These animate objects not only sense how they are being used, but also communicate with each other to develop a shared intent, and provide prompts and cues to encourage specific actions. In this way, the human-object-environment system creates affording situations in pursuit of shared intentions and goals. In order to determine when to provide prompts and cues, the objects need to have a model of how they ought to be used and what intention they are being used to achieve. Consequently, affordances become not only the means by which actions are encouraged but also the manner in which intentions are identified and agreed.

## Introduction

This paper is motivated by three simple questions: (i) how do people know how to use smart objects (i.e., how do people respond to the form and function of smart objects in order to achieve goals)? (ii) how do objects make sense of the manner in which they are being used (i.e., can objects recognize different ways in which a person interacts with them)? (iii) how should designers design smart objects to enable people to use these appropriately (i.e., is it possible to better inform design practice so that we can predict the successes and challenges of interacting with smart objects)? Unpacking this a little, a “smart object” (Kortuem et al., 2010) is some artifact with which a person can interact, but which is capable of sensing that it is being interacted with, capable of making inferences from these sensor data, capable of communicating these inferences with other artifacts, and capable of guiding the person to perform further actions. Knowing how to use an object *could* involve problem-solving in which features of the object are associated with functions, and these functions associated with a plan to act. But often, there is little overt, conscious awareness in performing the action. By way of a motivating example, imagine that you are reaching to pick up a cup containing a hot drink.The handle of the cup could be grasped in a particular way (say, two fingers through the handle and the thumb resting on the top), or the body of the cup could be held in your palm with fingers and thumb wrapping around it. Which grasp you select depends on, among other things, the heat of the contents of the cup, whether the cup is full to the brim, whether the handle is on one side or the other. However, it is unlikely that your selection arises from conscious deliberation: you simply pick up the cup. As Wittgenstein noted, “The aspects of things that are most important for us are hidden because of their simplicity and familiarity.” (Wittgenstein, 1958, p.50). The concept of affordance helps frame this activity and explain how it can be performed without conscious intervention. In other words, very often, we simply “know” what to do. From the perspective of cognitive psychology, this knowledge has been termed “procedural” (Anderson, 1981), tacit (Polyani, 1966), “implicit” (Berry and Broadbent, 1988), or “automatic” (Schneider and Shiffrin, 1977; Shiffrin and Schneider, 1977). It is from these different traditions that one can appreciate what “affordance” involves. Relating this to design, one could also suggest that understanding such implicit, subliminal interaction could align neatly with some formulations of the concept of nudging, in which people might be encouraged to perform actions on certain ways and where such encouragement would be at the edge of conscious awareness (Thaler and Sunstein, 2008). Nudging could, for example, involve the cup prompting the user to pick it up (perhaps to encourage the person to drink more water) or it could encourage picking the cup up with one hand rather than the other (perhaps as part of rehabilitation) or it could encourage the person not to pick it up (perhaps to discourage the person from drinking coffee after a certain time of day). In these instances, the cup takes on the role of a smart (possibly irritating, possibly helpful) partner in performing an action. For me, the question is whether this partnering could be both beneficial and performed without conscious awareness. So, could interaction with a smart object be described in terms of affordance. I begin this paper with a short account of how the concept of affordance has developed, with particular reference to interaction design.

### A brief history of “affordance”

For many people working in the field of Human-Computer Interaction (HCI), their first encounter with the concept of “affordance” probably came from Norman's (1988) *The Psychology of Everyday Things* [he later, in 2002, rewrote this as *The Design of Everyday Things*]. In this book, Norman presents “affordance” as an act of interpretation, in which the form of an object is seen in relation to a specific action. So, the flat plate on a door “affords” pushing. From this perspective, the “affordance” is a visual clue, provided by the object, as to its intended functioning: “Plates are for pushing. Knobs are for turning. Slots are for inserting things into. Balls are for throwing or bouncing.” (p. 9). What is deceptively attractive about this notion, for design at least, is the implication that the physical form of the object corresponds with a conceptual model that the user of the object brings to the interaction. In other words, Norman's (1988) definition, while it looks to be based on perception, is really about interpreting the object's functions in terms of specific features, and linking this interpretation to a goal that one wishes to achieve. Returning to our example of picking up a cup, this implies that one needs to selectively determine which features of the cup (and its contents) are most salient to the goal of drinking from it (under certain constraints, like not spilling the contents or scalding one's hand). In other words, there is an implication that, prior to performing an action, one engages in a sort of problem-solving which allows salient features to be elicited and interpreted. Later, Norman (1999) distinguished “perceived affordances” from what he defined as Gibsonian or “real affordances.” It is worth noting at this point that there are extreme differences between “perceived” and “real” affordances. For one thing, Gibson's (1977, 1979) claim is that we have a perceptual system which is tuned (through evolution, experience, learning) to the environment. This means that there is no requirement for any form of interpretation of information; we just “see” (or hear or otherwise perceive) a pattern to which we can respond. To repeat our example, a cup full of steaming hot coffee is “seen” as a different object (supporting different actions) to a half-full cup of cold milk. When Norman uses the word “perceive,” this is not in the same manner that Gibson uses it; Norman seems to suggest that perception is an active process of extracting features and assigning meaning, whereas for Gibson, perception is the capability of being sensitive to information. Later still, Norman (2008) separated “real affordances” from signifiers, i.e., perceptual information about objects.

It is worth tracking the term “affordance” back further. Gibson taught a course on the phenomenological philosophy of Merleau-Ponty, and the Gestalt psychologist Koffka was a colleague of Gibson's in the 1930s (Kaufer and Chemero, 2015). Key to Merleau-Ponty's (1945) Phenomenology is the notion of *intentionality*, which is concerned with how we “see” an object in terms of how we will interact with it (rather than as a collection of features). That is, we see the *intentional object* in relation to our intended action. One way of appreciating this, is through the concept of “Gestalt” (with which Merleau-Ponty was familiar), which is not some property of the object but rather the combination of the sensory stimulation evoked by an object in a given context. In Norman's (1988) glossing of “affordance,” the object becomes imbued with meaning in a way that Gibson (and Gestalt psychologists, and Merleau-Ponty) resisted. This means not only that the “Gestalt” is more than the sum of its parts, but also that the object can be interacted with differently under different conditions. This reiterates our distinction between cups of hot coffee and cold milk. In order to interact with an object, the individual must have the ability to act upon or with that object; and so, the individual can be considered in terms of effectivities (Turvey and Shaw, 1979). In this respect, environmental constraints (in terms of properties of objects) are responded to in terms of bodily constraints (in terms of effectivities). Stoffregen (2003) and (Chemero, 2003; Chemero et al., 2003) dispute the implication that “affordance” arises because the object elicits a dispositional response in the user, and they propose that this should not be regarded in terms of dispositions (that is, consistent responses to objects) but rather in terms of abilities (that is, flexible and adaptive styles of interaction). Furthermore, as Osiurak et al. (2017) point out, the notion of effectivity conflates two kinds of action possibilities—those offered by the body and those provided by objects.

In terms of Gestalt psychology, Lewin (1936) developed the concept of *Aufforderungscharaktere* (translated as “demand character,” “invitation-character” or “prompt-character”) indicating the properties of an object which call for a certain behavior. This describes interaction with an object in a context, in terms of “valences” (which are a function of the person's (motivational) state and the properties of the environment in which they are acting). This implies (I feel) that the relationship between object and action would vary according to person and environment (much as Merleau-Ponty, 1945 suggests). In contrast, Gibson (1979) claims that “affordances” are invariant and quotes his colleague Koffka as saying, “*Each things say what it is…a fruit says ‘Eat me’, water says ‘Drink me’, thunder says ‘Fear me’…*” (Koffka, 1955, p.7). My problem with this claim is that it seems to return us to the idea that an “affordance” is a property of the object and is independent of the viewer. In contrast, in order to perceive an object's affordance, one needs to have prior experience of using objects of this type and a set of beliefs as to how such objects ought to be used. This gives a strong cultural and experiential basis to the response to affordance in ways that Gibson was seeking to avoid through his insistence that perception of affordance was a direct response to the visual appearance of an object. Gaver (1991) suggested that one could separate affordance from perceptual information, and introduced terms such as “false affordance” (in which the form of object implies a possible action, say a decal on a product that looks like a button) and “hidden affordance” (in which perceptual information is obscured). Although the notions of “false” and “hidden” affordance are useful, this relies on the conflation of “affordance” with function. This creates further confusion in the application of affordance to design—should we be concerned with designing visual signifiers that cue an action (which is, surely, much the same as stating that the form of an object signifies its functions, which designers know anyway) or does affordance provide another perspective on design?

From his interpretation of Gibson's various proposals about “affordance,” Chemero (2009) suggests that, “Affordances are neither properties of the animal alone nor properties of the environment alone. Instead, they are relations between the abilities of an animal and some feature of a situation.” (p.191). This observation is significant to the current paper for three reasons. First, it recognizes that affordances arise through relations in animal-object-environment systems (rather than existing as properties of any constituent component). This raises questions about what the designer is designing in order to support affordance. My answer to this is that design, in this context is less about the fashioning of objects (although, of course, these are important) and more about choreographing situations in which people interact with objects. Second, the idea that affordances are relations implies that people rarely attend to the specific features of the context in which these relations occur. In their discussion of affordance, Still and Dark (2013) suggest that people respond to affordances “automatically,” i.e., with little or no conscious awareness or need for attentional control. Similarly, the use of highly familiar objects would involve minimal attentional demand, but when confronted with a novel or unfamiliar object, there would be a need to construct a plan of how to interact with it (Humphreys, 2001; Humphreys et al., 2010). Furthermore, if affordances guide action then this could only be for someone able to perceive the relevant “information,” able to perform the relevant action, and able to relate the action to a desirable goal (Roux and Bril, 2005; Fairlie and Barham, 2016). As Kirsh (2013) has it, “goals make perception enactive” (Kirsh, 2013, p. 10). To illustrate this, he gives the example of a stonemason (or bricklayer) who “…will look at bricks for places to apply cement; when looking at an odd brick he will ‘see’ the particular trowel shape that is needed.” (Kirsh, 2013, p. 9). For someone without the experience of bricklaying, there is less likely to be distinctions between bricks and less likely for these distinctions to result in changes in action.

### Formally describing affordance

Lewin (1936), who we have already noted as a providing a precursor definition of what became known as “affordance,” developed a simple equation (Equation 1) to model behavior (*B*) as a function *f* of Person (*P*) and environment (*E*).

(1) $$\begin{array}{r} B=f\left(P,\;E\right) \end{array}$$

This simply states that behavior of a person is directly connected to the environment in which they act. In order to address some of the issues surrounding the debate over what “affordance” might be, Turvey (1992) proposed a formal definition (Equation 2 which one can see is inheriting Lewin's idea). This can be expressed as:

(2) $$\begin{array}{r} W_{p,\;q}=j\left(X_{p},\;Z_{q}\right)\;possesses\;r \end{array}$$

In other words an Environment or World, *W*, has properties p and q which can be defined as the joining, j, of an object *X* (with property *p*) and an animal *Z* (with effectivity *q*) in order to produce an affordance relationship, *r*. In this account, the animal has a set of dispositions, characterized in terms of effectivity, which enable it to respond to object properties. So, an adult human hand can grasp the handle of a full cup and lift it in a way that a child's smaller hand might not be able to: from Equation (2), the cup_handle (for the adult) affords grasping (because its property, *p*, defined by its size and shape, matches the disposition, *q*, of the person, defined by hand-size), and the full_cup affords lifting because of the adult's strength. As noted previously, Stoffregen (2003) questioned Turvey's (1992) claim that effectivities are dispositions. He suggested that it makes more sense to regard these as abilities that can be called upon in a given situation. This is useful because it means that the response that a designer might expect to elicit using a given form could be correct in terms of effectivity but not ability, and so, affordance is about matching ability not disposition.

For Stoffregen (2003), affordance emerges from the World-Object-Animal system and is not a property of any one of these in isolation. Thus, Stoffregen (2003) offered Equation (3).

(3) $$\begin{array}{r} W_{p,\;q}=\left(X_{p},\;Z_{q}\right)\;possesses\;h \end{array}$$

What the formal descriptions struggle to present is the discretion with which such responses are made. In other words, is it possible to not respond to an object's “solicitation” of a response? Certainly, this is not easy to see from Turvey's (1992) account. For Stoffregen (2003), the post-hoc description of an affordance as something that has occurred in a system, rather blurs this problem.

To consider the problem more concretely, the notion of Stimulus-Response Compatibility has been a staple part of Ergonomics design thinking for the past half century. To illustrate this idea, imagine that you have a row of 4 lights in front of you (labeled 1–4), and between you and the lights is a row of 4 buttons (labeled A–D). The buttons and lights are arranged so that 1 and A are adjacent, etc. When one of the lights turns on, you must press one of the buttons to turn off this light as quickly as possible. In the adjacent (or congruent) arrangement, when light1 turns on, you press button A. In an incongruent arrangement, when light 1 turns on, you have to press, say, button C. Not surprisingly, the congruent arrangement leads to much faster performance. Early accounts of the SRC suggested that the performance differences were due to “translation” (Fitts and Seeger, 1953; Fitts and Deininger, 1954; Welford, 1976). People prefer arrangements in which the elements (light and button) are congruent, and this is termed a Population Stereotype (there is some work to suggest that different cultures might have slightly different Population Stereotypes). Furthermore, most people produce faster responses with fewer errors in Sets of stimulus-response pairings which have this preferred arrangement, and this defines Stimulus-Response Compatibility (SRC). A popular explanation of SRC relates to the ability to extract salient features and pair these with an appropriate response. This is the “dimensional overlap” model (Kornbulm et al., 1990) and broadly contrasts the overlap of dimensions (elements) in a set (i.e., the congruence of arrangements) with the relevance of elements within a set (i.e., how the features of a stimulus relate to a response). For example, button presses could conceivably be made in response to proper names. In this case, there is no overlap between the layout of the buttons and the nature of the stimulus, and there is no relevance of stimulus content to response. On the other hand, button presses might be to the lights (which might be labeled with proper names). In this case, there is no relevance of the names, but there might be overlap between the position of the light and the position of the button. Finally, the congruent condition (arranging buttons and lights as described earlier) has both overlap and relevance.

The relevance of SRC to HCI has been recently reviewed in a paper by Proctor and Vu (2016), and they suggest that it continues to provide useful guidelines for design. There is much to be said for the empirical evidence from SRC. From the perspective of affordance, it could be argued that SRC arises when information from environment (stimulus) relates to ability (response). In other words, there is potential argument that removes the need to appeal to a “translation” or a “dimensional overlap” to explain this. In their paper, Proctor and Vu (2016) argue against “affordance” and suggest that it merely describes a particular form of spatial compatibility. I felt that they misrepresented the basic ideas of affordance and agree with Stins and Michaels (1997), who argued that, in SRC studies, the “information” could include more than just the position of the response buttons (as SRC tends to assume). Crossing one's hands in SRC experiments leads to an increase in reaction time, even when the position of stimulus and response objects remain constant, and this does not seem to be the result of a simple biomechanical constraint; reactions using crossed hands cannot be explained solely by conflict management, as proposed by the dimensional overlap model. This suggests that the relationship between response and stimulus involves more than the simple mappings that SRC assumes. Further, SRC studies often fail to control properly for the different compatibility effects that could arise from the use of different response actions that are required. Finally, SRC studies do not seem to be able to account for how changes in ability can lead to changes in performance. Having said that, the formal approaches to affordance outlined, above do not account for this either. If we refer back to the formalisms outlined in Equations (2) and (3), it is difficult to see how these could account for the differences in SRC. In both congruent and incongruent conditions, X_p_ would be “light on,” and Z_q_ would be “press button.” So, perhaps, we need to elaborate the X_p_ description to include X_p1_ “light on” + X_p2_ “light adjacent to button” (in the congruent condition), and to elaborate Z_q1_ “associate light label with button label” + Z_q2_ “press button” in the incongruent condition.

While the formal descriptions of Turvey (1992) and Stoffregen (2003) are directed at the immediate relationship between an object and its user, this does not fully capture the situation in which the relationship arises. For Kirlik (2004), a problem with Stoffregen's (2003) equation is that there does not appear to any constraint on how to define the parameters. Abbate and Bass (2017) develop a variation of Stoffregen's (2003) formalism that works with *a priori* constraints (Equation 4):

(4) $$\begin{array}{r} Possesses\left(affordance_{i}\right)\left(X_{p},\;Z_{q}\right) \end{array}$$

This relationship becomes expandable with specific values of the elements of *X* that are relevant to a given “goal” and with specific values that define the ability of *Z* required to respond to these features. As an example, Abbate and Bass (2017) propose that an aircraft cabin door is plugged into its fitting under high external pressure, and that (on the ground) the door can be opened by pulling out a lever and then turning it. So, in this case, there are two affordances of interest, i.e., *leverLiftable*, and *doorOpenable*. These can be defined as follows:

*possesses(leverLiftable)(X*_*p*_*, Z*_*q*_*)* = *true if:*

*X*_*p*_*.Airspace.Aircraft.Cabin.Door.Lever*_*p1*_*[Slot][bottom_of]* = *overlapping* ∧

*X*_*p*_*.Airspace.Aircraft.Cabin.Door*._*p1*_ *x (X*_*p*_*.Airspace.Aircraft.Cabin*._*p1*_ *- X*_*p*_*.Airspace*_*p1*_*)* + *X*_*p*_*.Airspace.Aircraft.Cabin.Door.Lever*_*p2*_ ≤

*Z*_*q*_*.Airspace.Aircraft.Cabin.Door.Lever*_*q1*_*[position_up]*

*possesses(doorOpenable)(X*_*p*_*, Z*_*q*_*)* = *true if:*

*X*_*p*_*.Airspace.Aircraft.Cabin.Door.Lever*_*p*1_*[Slot][top_of]* = *overlapping* ∧

*X*_*p*_*.Airspace.Aircraft.Cabin.Door*._*p2*_ *[Cabin][left_of]* = *contained_within* ∧

*Z*_*q*_*.Airspace.Aircraft.Cabin.Door*._*q1*_*[position_back]* = *true* ∧ *Z*_*q*_*.Airspace.Aircraft.Cabin.Door*._*q1*_*[translate_left]* = *true*

This formal description elaborates the context under which the lever “affords” lifting and the door “affords” opening (in terms of external air pressure and the position of the lever, and in terms of the action performed by the person). In order for the person to perform the action, they need to apply the appropriate force to the lever—so this is intended to reflect ability rather than disposition. However, there is something missing from these formal accounts, and that is the rationale for performing the action in the first place. One way of considering this is to turn to suggestions that “affordance” is hierarchical and can be described in terms of different levels.

### Levels of affordance

Although Abbate and Bass (2017) relate values for X and Z to an affordance relationship, they do not say how the affordance itself relates to a particular “goal,” such as lift_lever or open_door. McGrenere and Ho (2000) use the term “possibility for action” to indicate that there might be levels of affordance. One way of thinking of this is in terms of “sequential affordance” (Gaver, 1996). For example, grasping a lever handle “affords” lifting, which then releases the door and, so “affords,” opening the door. In this sequence, affordances are “nested,” i.e., the lever's “graspability” is nested in the door's “openability.” I am not convinced that it makes sense to call this a “sequence of affordances,” so much as a sequence of actions, but can see how one could apply the formal descriptions outlined above to each “state” in the ongoing sequence of interactions between person and object. What is interesting about this perspective is that the “door_handle” contributes to several “affording situations.” Consider, for example, turning the door handle when you were carrying a pile of books or a cup of coffee, as opposed to turning it with an unencumbered hand.

The notion that affordances could have multiple instances was also discussed by Hartson (2003) who suggested that affordances could be: cognitive, physical, sensory, with each of these helping users to perform cognitive, physical or sensory action. This seems to me to conflate different notions of “affordance” in ways that are not helpful. For instance, while affordance describes the relationship between the form of an object and the person's action, it is not obvious how this relates to cognitive and sensory actions. Similarly, Turner (2005) contrasted what he termed “simple affordance” (which draws on Gibson's definition) with “complex affordance” (which involves interpretation and response to an object's form in terms of the user's culture, history, praxis). However, applying the term “affordance” to such different behaviors can only serve to increase confusion. To this end, I proposed a different terminology to describe these different levels.

### Forms of engagement

**Figure 1:**
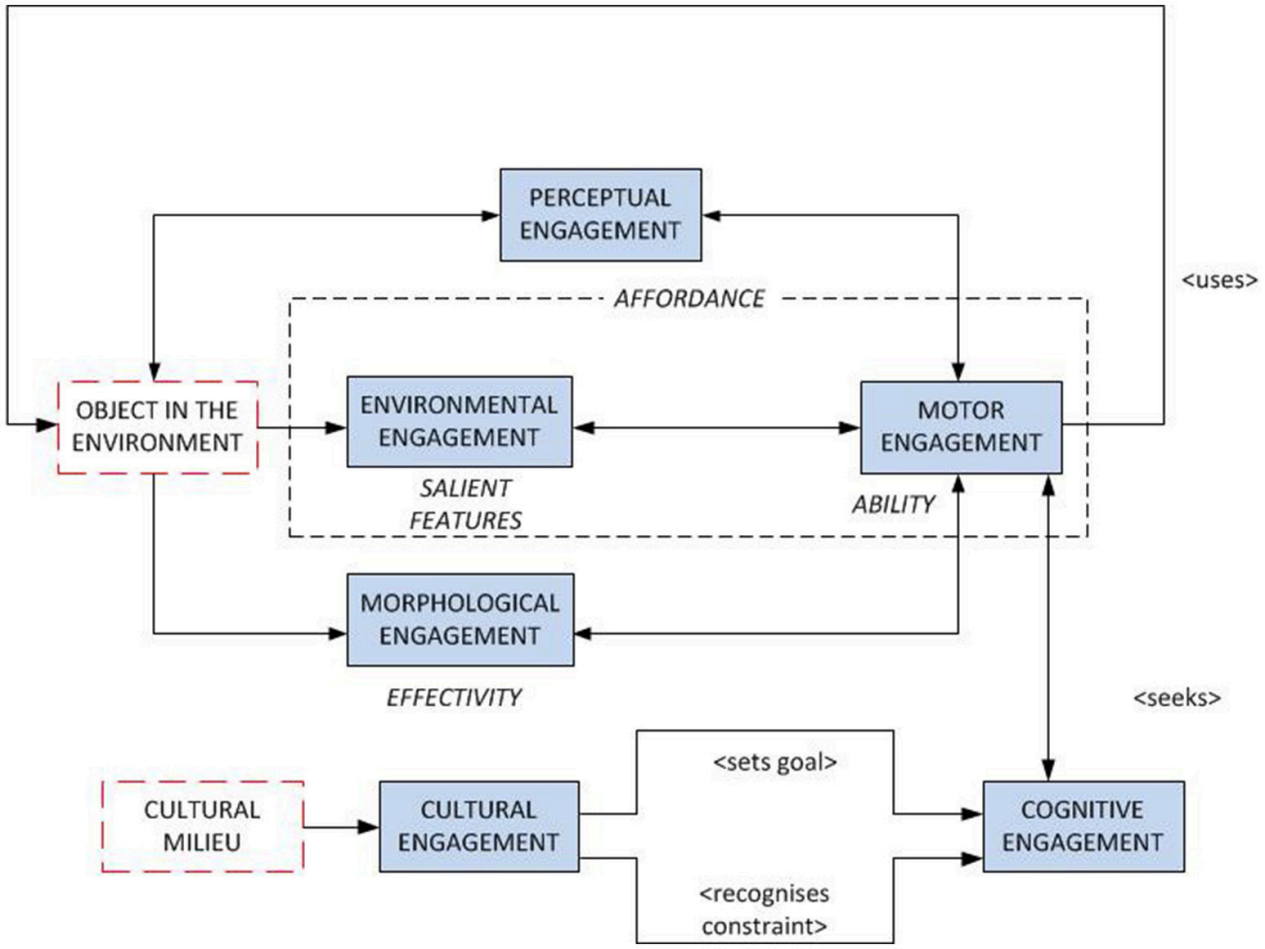
Forms of Engagement (2017 version).

In order to explore the concept of affordance further, and to make use of the suggestion that there are different levels of “affordance” that provide constraints of the ways in which we interact with objects, I developed the idea of forms of engagement (Baber, 2003, 2006). In this, the focus is on the ways in which we engage with objects and how different forms can serve to support and constrain each other. The most recent version of this concept is illustrated by Figure 1. The arrows are intended to indicate the relation “constrains.” Note that, at the center of Figure 1 is a dotted box which is labeled “affordance.” This describes a relationship between the ability to recognize salient features in an object (Environmental Engagement) and the ability to act using that object (Motor Engagement).

Figure 1 separates the effectivity of the person, in terms of *Morphological Engagement*, from ability, in terms of motor engagement. There are two reasons for this: first, morphology is partly dispositional, e.g., in terms of the size of the hand; and second, hand shaping will be influenced by subsequent actions, e.g., when reaching to grasp an object, hand shape is modified in anticipation of the type of grip required to respond to properties of the object, such as weight, fullness, slipperiness etc. (Wing et al., 1986), and this will also be influenced by *Motor Engagement*, i.e., Rosenbaum et al. (1992) notion of “end-state comfort” explains why people might adopt an uncomfortable grip at the beginning of an action, in order to end an action with a comfortable grip. For example, if a wine glass is upside down on the table, you will probably twist the hand awkwardly to pick it up in order to turn it right-way up. So, there are a limited set of ways in which an object can be grasped by the human hand and the selection of grasp combines object properties with intended movements. That is, a hand of a given size will have limits of how it can grasp objects, but *how* the grasp is performed reflects the ability and intentions of the person, which will vary according to a host of situational factors, as well as prior experience.

In order to act on an object, there is a need to respond to the “information” that it conveys. I am using the word information in a Gibsonian sense, and apply the term *Environmental Engagement* to reflect this. Consequently, an affordance arises as the result of the relationship between *Environmental* and *Motor Engagement*. For example, people can make rapid judgements about whether to turn their body to fit through narrow apertures as they approach these (Warren and Whang, 1987) and can make such judgements even when their bodies have been modified to an unfamiliar size, e.g., when wearing “pregnancy packs” on the front (Franchak and Adolph, 2014), or when wearing rugby shoulder pads (Higuchi et al., 2011). Furthermore, increasing the weight of the body, e.g., by wearing a heavy rucksack, can alter judgements of the steepness of a hill (Profitt, 2006). The implication is that there is a “body-scaled” perception of some features of the environment that can guide some actions (Warren, 1984; Fajen, 2007). In other words, people are able to “see” aspects of the object, or the environment, in terms of an action that they both want, and are able, to perform. We can directly relate this proposal to Equation (2), e.g., imagine we are interested in stair-climbing, and the property of the world, *Xp*, is the height of a stair riser, and the property of the person, *Zq*, is their leg length.

This could, of course, be termed “perception-action coupling” (which is a common expression of Gibson's ideas and a reasonable explanation of affordance from the perspective adopted in this paper). So, I retain the term “affordance” for the specific relationship between object and action—and regard this as an emerging property of the world-object-person system. However, this relationship is bounded by the other forms of engagement. The suggestion that *Motor Engagement* is directed toward subsequent action implies an intention, but I argue that there is equal scope that the “intention” can be defined in response to the *Motor Engagement* (opportunistic or situated action). At the very least, there is a two-way exchange between the action-as-performed and the goal-state of that action. The role of *Cognitive Engagement* is to provide this high-level management on ongoing actions. Across the various forms of engagement, *Perceptual Engagement* relates salient features to changing state of the object-person system. Finally, the notion of an “acceptable” goal could relate to the culture in which one is acting. This *Cultural Engagement* relates to the idea of “complex affordance” (Turner, 2005). It could also relate to the concept of “cultural affordances” developed by Ramstead et al. (2016).

The basic concept of Forms of Engagement is intended to retain “affordance” as a simple relationship between the actions a person performs to the features of the object that they are using. The connections between the different forms represent the constraints that shape and respond to this relationship. I claim that this provides a useful way of conceptualizing interaction, and use this to explore ways in which one can design animate objects.

### Animate objects

Having proposed that interaction comprises a number of Forms of Engagement, one can relate these to the possible inferences that animate objects could make as they are being interacted with. At the most basic level, sensors on the object could provide data to characterize the motion, orientation, position, etc. of the object. However, what would be most useful is not just identifying that a movement has been made but also to identify how well that movement has been made, e.g., has it been performed smoothly, hesitantly, with tremor etc. In this way, the object would be able to make inferences about the user's Motor Engagement and abilities. Additional sensing capability could be added to monitor hand shape and movement as it approaches the object, in order to make inferences concerning Morphological Engagement. This could be used to determine the type of action that the person might be intending to make, even before picking up or handling the object. Previously I have contrasted these as epistemic or ergotic gestures, to reflect the fact that such actions could be treated as “gestures” which have the intention of altering the state of the user's environment (Baber, 2014).

The object, assuming that it can modify its appearance, could encourage Environmental Engagement through changes that emphasize specific features. So, when a handle rises on the side of a cup, people are more likely to use the hand on that side of the cup to pick it up (Baber et al., 2017). Having some knowledge of where the object is being used could also influence the definition of appropriate actions, through Cultural Engagement. Combining inferences drawn from Motor and Morphological Engagement, the object could infer the most likely intention of the person, and use this inference to provide additional cues and guidance (Jean-Baptiste et al., 2015).

Let us assume that the “smart object” looks like something familiar, say a cup, which has been fitted with sensors (Gellersen et al., 1999; Baber et al., 2017). On the one hand, this is an object that we “know” how to use, but on the other hand, this is an alien object that is capable of doing things that we do not, necessarily, fully understand. The cup could, for example, be part of a system that monitors our daily liquid intake and the system could have a “goal” of ensuring that we drink a specified quantity of liquid, or it might be part of a system that has the “goal” of reducing our caffeine intake. One way in which such “goals” could be communicated to the user would for the artifacts themselves (through lights, sounds, movement etc.) to provide feedback and prompts to the person. In this way, the form of the objects could display their function. I am interested in this relationship between form and function (both in terms of “normal” and “smart” objects), and how the “function” of an object corresponds to the action in which it is used. There are many instances in which the “action” is quite different from the designed “function,” e.g., a laptop could be used to prop the leg of a wobbly desk, or as a tray to carry several coffee cups, or as a weapon.

### Implications for design

I close this paper with some observations on how the concept of Forms of Engagement could apply to broader areas of HCI design. There seems to me to be a division between those practitioners who are interested in usability and those interested in user experience (Baber, 2015). The “usability” focus tends to emphasize performance (although, of course, International Standards Organization definitions of usability include efficiency, effectiveness *and* experience), while “user experience” tends to focus on the emotional response (from pleasure to frustration) that users get from their interactions with technology. Broadly, I would suggest that usability takes as its “context of use,” the region in Figure 1 that is defined by Environmental, Motor, Morphological, Perceptual and Cognitive Engagement, while “user experience” takes as its focus the region in Figure 1 that is defined by Cultural, Cognitive and Perceptual Engagement. Of course, I am not claiming that there is not overlap between these regions, but it seems to me that the differences in practice relate to the different levels of analysis that practitioners emphasize. It would, one hopes, be profitable and useful to merge these practices of evaluation of HCI.

A final point for this paper is that I do not believe that it is possible to “design affordance” into an object. This is the fundamental argument made in this paper. However, I do believe that it is possible to create affording situations—and that this is what good design has always sought to achieve. Knowing how a person with given ability would interact with an object to achieve a given goal in a given context is central to ISO definitions of Human-Centred Design. What I have offered in this paper is a conceptual framework that illustrates this goal, and relates it to an unambiguous interpretation of the concept of “affordance.”

## Author contributions

The author confirms being the sole contributor of this work and approved it for publication.

### Conflict of interest statement

The author declares that the research was conducted in the absence of any commercial or financial relationships that could be construed as a potential conflict of interest.
